# Hairy cell leukemia: A diagnosis by endoscopic ultrasound guided fine needle aspiration

**DOI:** 10.1186/1742-6413-3-1

**Published:** 2006-01-23

**Authors:** Regina S Meara, Vishnu Reddy, Juan Pablo Arnoletti, Darshana Jhala, Shyam Varadarajulu, Nirag Jhala

**Affiliations:** 1 Department of Pathology, University of Alabama at Birmingham, Birmingham, AL, USA; 2 Department of Surgery, University of Alabama at Birmingham, Birmingham, AL, USA; 3 Department of Gastroenterology, University of Alabama at Birmingham, Birmingham, AL, USA

## Abstract

**Background:**

Endosonography (EUS) guided FNA is a relatively new imaging modality which is increasingly used for sampling deep-seated lymph nodes in the diagnosis and staging of various malignancies, both primary as well as metastatic. It is also useful for staging of non-Hodgkin's lymphoma as well as diagnosing recurrence. The diagnosis of leukemia on FNA samples from deep-seated lymphadenopathy poses an even greater challenge. Hairy cell leukemia (HCL) is an uncommon, but distinct, lympho-proliferative disorder of B cell origin. It usually affects the spleen and bone marrow and uncommonly involves lymph nodes. There are only a few cases reported where HCL was diagnosed on FNA specimens.

**Case presentation:**

We report the first case of HCL accurately rendered on EUS-FNA samples.

**Conclusion:**

This report underscores the concept that the presence of a cytopathologist in the endoscopy suite plays an important role in providing accurate diagnoses of lymphoid lesions biopsied with EUS-FNA.

## Background

Image-guided fine needle aspiration (FNA) of lymph nodes is commonly used for detecting and staging malignancies and is also useful in demonstrating therapy-associated change [1,2]. Despite concerns[3], there is an overwhelming consensus amongst cytopathologists that with correct utilization of the technique, cytology in conjunction with immunophenotyping can serve as a powerful modality to diagnose as well as subtype lymphomas in majority of cases [4-10]. EUS guided FNA may provide a high cellular yield even from small (<25 mm) lymph nodes [11]. This modality can also provide sufficient material to make a morphologic diagnosis and in addition enough cells to perform ancillary studies [1,2,11,12].

Leukemic infiltrates in solid organs and lymph nodes are rare and pose an even greater challenge than diagnosing lymphoma on cytology samples. Hairy cell leukemia (HCL) is an uncommon, but distinct, lympho-proliferative disorder of B cell origin. It usually affects the spleen and bone marrow and uncommonly affects lymph nodes [13,14]. There are only a few cases reported in which HCL was diagnosed on FNA samples [15-17]. The use of EUS-FNA in the diagnosis of leukemia has not been reported. Here we report an unusual case of HCL diagnosed from samples obtained by EUS-FNA.

## Case presentation

A 79-year-old white male with a history of HCL for which he underwent splenectomy 19 years ago, presented with acute onset of abdominal pain, nausea and vomiting. These symptoms resolved spontaneously after a day or two. The patient denied fever, night sweats, weight loss, dysphasia, skin rashes, hemetemesis or altered bowel habits. Physical examination revealed a soft, nontender abdomen and no palpable peripheral lymphadenopathy. The patient had been treated with 2-chlorodeoxyadenosine (2-Cda) therapy for the past four years.

### Laboratory values

The patient's white blood cell count remained within the reference range for the past 4 years, ranging from 4.8–6.5 × 10^3^/μl (reference range: 3.5 – 10.0 × 10^3 ^μl). Lymphocytes ranged from 47–53% (reference range: 15–52%). No atypical lymphoid cells were noted in the peripheral smears.

### Imaging Studies

A CT scan of the chest, abdomen and pelvis demonstrated multiple enlarged mediastinal lymph nodes as well as bulky retroperitoneal and gastro-hepatic lymphadenopathy suggestive of lymphoma.

### EUS- FNA

After informed consent, EUS was performed which demonstrated multiple enlarged lymph nodes around the gastro-hepatic region, the largest of which measured 14 × 8 mm. The lymph node appeared hypoechoic with round borders.

### Cytology

**Figure 1:**
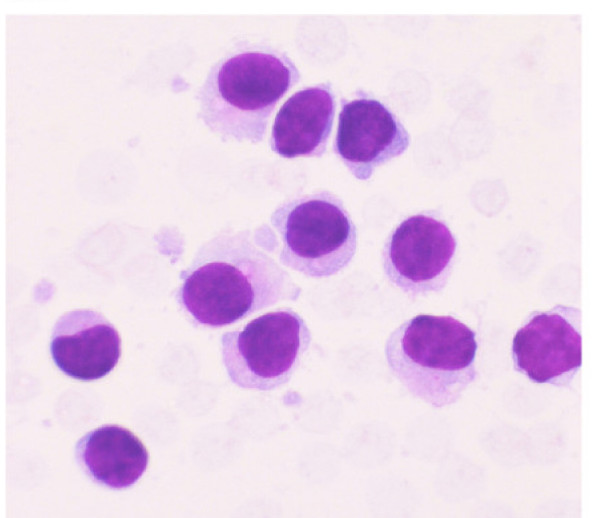
Aspirate from the EUS-FNA of lymph node revealed small to intermediate sized mononuclear cells with moderate cytoplasm, round to oval nuclei with smooth nuclear borders, a stippled chromatin pattern and occasional single nucleoli. The cytoplasm is pale grey with hair-like and short, blunt, cytoplasmic projections. (Stain: Diff Quik, Magnification: × 40).

**Figure 2:**
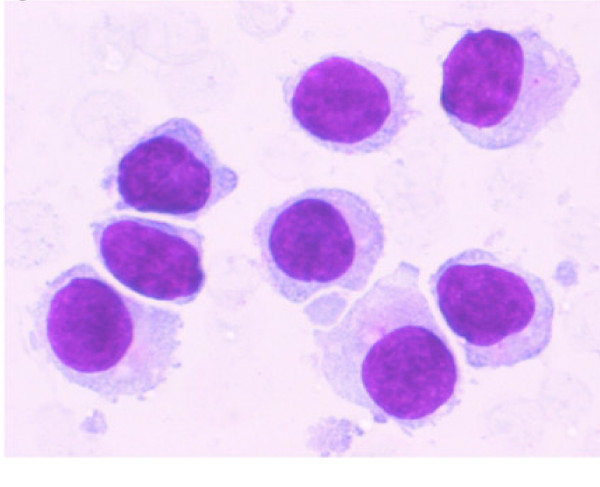
A higher magnification of the aspirate from same area as shown in Figure 1 shows small to intermediate sized mononuclear cells with moderate cytoplasm, round to oval nuclei with smooth nuclear borders, a stippled chromatin pattern and occasional single nucleoli. The cytoplasm is pale grey and granular with hair-like and short, blunt, cytoplasmic projections. (Stain: Diff Quik, Magnification: × 60).

Air-dried smears from samples obtained by EUS-FNA were stained with Romanowsky stain (Diff Quik) in the endoscopy suite to determine whether the target lesion was indeed aspirated as has been described previously [2]. Aspirates revealed small to intermediate sized mononuclear cells with moderate cytoplasm, round to oval nuclei with smooth nuclear borders, a stippled chromatin pattern and occasional single nucleoli. The cytoplasm was pale grey and granular with hair-like and short, blunt, cytoplasmic projections (cytoplasmic blebs) (figures 1, 2). Occasional groups of glandular cells with a honey-comb appearance, preserved nuclear to cytoplasmic ratio, regular nuclear membrane and lack of marked pleomorphism and mitoses helped distinguish them from the gastrointestinal tract mucosa as benign epithelial cell groups. Additional samples were collected in RPMI medium for flow cytometry examination to perform immunophenotyping and to determine clonality. Additional samples were collected in Hank's balanced salt solution for paraffin embedded cell block preparation. Several smears prepared in the endoscopy suite were fixed in 95% alcohol and were later stained with Papanicolaou stain.

### Flow cytometry results

**Figure 3:**
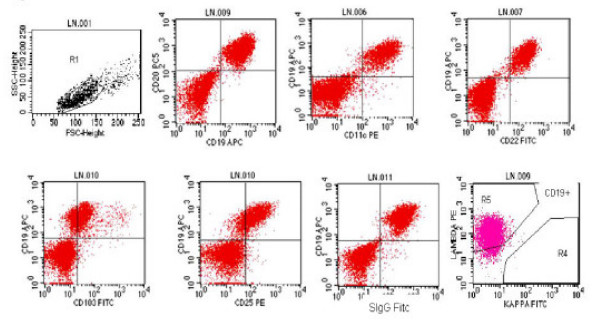
Immunophenotyping performed using selective gating on lymphoid cell population shows that neoplastic cells are positive for clonal B-cells (>98%) expressing CD19, CD20, CD11c bright, CD25 bright, CD103. surface immunoglobulin and are lambda light chain restricted.

The EUS-FNA sample was analyzed by 4 color flow cytometry (Facscan, BD^® ^Instrument, Becton Dickinson Inc., San Jose, CA). A total of 2.2 million mononuclear cells with viability of 89% are obtained. These cells are stained by panel of various fluorochrome conjugated antibodies (mouse anti-human monoclonal antibodies, dilutions ranging from 1:2 to 1:10, Caltag^® ^Laboratories, Burlingame, CA.,). After standard washing steps and fixation these are analyzed on BD^® ^instrument. Selective gating on lymphoid population (large cell gate) was positive for clonal B-cells (>98%) expressing CD19, CD20, CD11c bright, CD25 bright, CD103 and were lambda light chain restricted. CD10, CD5 and CD3 were negative (figure 3). This immuno-phenotype along with observed morphology on the flow Wright stained cytospin smear was also diagnostic of HCL. This immuno phenotype also ruled out other non -Hodgkin's lymphomas. A final diagnosis of recurrent HCL with involvement of the lymph node was made.

## Discussion

HCL is an uncommon, but distinct, lympho-proliferative disorder of B cell origin with an indolent clinical course. Less common sites of involvement by HCL include deep-seated lymph nodes, liver, bone, retroperitoneum, thyroid, etc. An accurate diagnosis of HCL depends on clinical features, as well as morphologic examination of peripheral blood smears, bone marrow and other tissues [14,18]. These patients generally present with anemia, neutropenia, monocytopenia, and splenomegaly [14,18]. Abdominal lymph adenopathy is unlikely to occur at the time of initial presentation; however, up to 15% of patients may develop abdominal lymphadenopathy during the course of their disease [14,19].

The presence of lymphadenopathy noted on the recent CT scan raised the suspicion of a non-Hodgkin's lymphoma. In a study by Goodman et al, second malignancies were noted in 22% (47 patients) of HCL patients who were followed for at least 7 years [20]. Only 3/47 (6%) patients who developed second malignancies developed non-Hodgkin's lymphoma [20]. Most cases of lymphoma developing in patients with HCL are diffuse large B cell lymphoma [18]. Occasional reports have also suggested transformation of HCL to other low grade B cell lymphomas [18].

EUS-FNA samples on rapid assessment in the present case revealed a small to intermediate-sized monotonous population of lymphoid cells; therefore, additional samples were collected for ancillary studies to rule out possible non-Hodgkin's lymphoma. The past medical history of HCL was not available to the cytopathologist at the time of aspiration. Morphology as well as a characteristic immunophenotype on flow cytometry confirmed the diagnosis of HCL and ruled out the possibility of other B cell lymphomas which could arise in a setting of HCL. This case is an example of how multi parameter (four color) flow cytometer could provide a characteristic immunophenotype of HCL even from a small quantity of cells (total of just over 2 million cells). The new generation of flow cytometers, for example BD Facscanto (Becton Dickinson, San Jose Ca) which has a capacity for eight-parameter detection, will offer an opportunity to provide immunophenotyping on even smaller quantities of neoplastic cells.

To the best of our knowledge, there have been only four previous reports where FNA was performed in patients with HCL (see table 1). In three of the four cases, a diagnosis of HCL was clinically suspected which helped establish the diagnosis of HCL on FNA samples [21]. In one unsuspected case of HCL, FNA did not prove to be as useful in making a diagnosis of HCL [21].

**Table 1:** FNA studies performed for Hairy Cell Leukemia in the literature

**Study**	**Year**	**FNA site**	**Percutaneous Vs. EUS**	**Flow cytometry**	**Primary Vs. Recurrent**	**Clinically suspected**
Moriarty A et al^1^	1993	Spleen	Percutaneous	Y	Primary	Y
Kaw Y et al^2^	1994	Mesentery	Percutaneous	Y	Recurrent	Y
Pinto G et al^3^	1995	Spleen	Percutaneous	N	Primary	Y
Lam et al^4^	1999	Thyroid	Percutaneous	N	Disseminated	N
Present Study	2005	Lymph node	EUS	Y	Recurrent	N

Unlike percutaneous FNA, EUS-FNA obtains samples by piercing the gastrointestinal tract mucosa. Rare fragments of glandular epithelium were noted in the aspirates in the present case; however, these did not pose any concern for metastatic carcinoma. Glandular epithelium can often be seen in the EUS-FNA samples, but their characteristic honey-comb appearance and benign appearing cytologic features are helpful in avoiding possible over-interpretations[2,22].

The patient in the present study was in complete remission with peripheral blood and differential counts remaining within the reference range. The patient had undergone splenectomy 19 years ago, and no parameters suggestive of recurrent HCL were noted in this patient. A recent review by Goodman, et al suggests that a complete remission has been noted in 50–95% of patients following 2-chlorodeoxyadenosine (2-Cda) therapy [20]. In a more recent study, 95% of 209 patients with HCL demonstrated complete remission after 2-Cda treatment; the median time of recurrence was 42 months [20]. In the present study, the patient was in remission for at least 4 years (48 months) following 2-Cda therapy and demonstrated abdominal pain and intra-abdominal lymphadenopathy as the first manifestation of recurrent disease.

## Conclusion

This case shows that a diagnosis of HCL can be accurately rendered on small EUS-FNA samples. This study also underscores the concept that the presence of a cytopathologist in the endoscopy suite is not only important to assess the specimen adequacy, but also plays an important role in obtaining additional samples for appropriate ancillary studies to arrive at an accurate diagnosis.

## Authors' contributions

All authors made substantial contributions to the intellectual content and/or presentation of the manuscript. RM (cytopathology fellow) is the first author, and she wrote the manuscript under the guidance of NJ (cytopathologist) senior author who coordinated the writing of this manuscript. VB (hematopathologist) and DJ (cytopathologist with expertise in hematopathology), helped with immunophenotyping as well as developing the manuscript. SV (endosonographer) performed EUS-FNA and provided necessary clinical information for writing and revising the manuscript. JPA (surgical oncologist) provided the necessary clinical information as well as follow up and revised the manuscript.
